# APOE-ε4 selectively modulates posteromedial cortex activity during scene perception and short-term memory in young healthy adults

**DOI:** 10.1038/srep16322

**Published:** 2015-11-10

**Authors:** J. P. Shine, C. J. Hodgetts, M. Postans, A. D. Lawrence, K. S. Graham

**Affiliations:** 1 School of Psychology and Cardiff University Brain Research Imaging Centre (CUBRIC), Cardiff University, UK, CF10 3AT

## Abstract

Apolipoprotein E (APOE) ε4 is a major genetic risk factor for Alzheimer’s disease (AD), yet the mechanisms by which APOE-ε4 influences early-life brain function, and hence, in turn, risk for later-life AD, are poorly understood. Here, we report a novel, and selective, pattern of functional brain activity alteration in healthy young adult human APOE-ε4 carriers. Our findings suggest that APOE-ε4 may influence vulnerability to poorer later life cognitive health via its effect on posteromedial cortex (PMC), a hub region within a brain network involved in spatial processing, and necessary for episodic memory. In two neuroimaging tasks, APOE-ε4 carriers showed an inability to effectively modulate PMC during scene, but not face and object, working memory and perception. This striking pattern overlaps both functionally and topographically, with the earliest cognitive deficits seen in clinical AD, as well as reported alterations in the default network in amyloid-positive individuals at increased risk of AD.

Some of the earliest and most consistent metabolic changes in Alzheimer’s disease (AD) are evident in posteromedial cortex (PMC), which comprises posterior cingulate, precuneus and retrosplenial cortices^1^,^2^. PMC forms part of the ‘default network’ (DN), a large-scale brain system more active during rest and minimally demanding tasks, but also implicated in episodic memory^3^. Specifically, PMC *deactivations* during memory encoding predict PMC *activations* elicited during memory retrieval^4^.

Consistent with AD pathology, PMC shows altered activity and connectivity in Mild Cognitive Impairment (MCI)^5^ and AD^6^,^7^; this pattern is also evident in asymptomatic amyloid (Aβ) positive older participants^8^,^9^. Similarly, older-aged individuals at increased genetic risk of AD (APOE-ε4 allele carriers)^10^ show disrupted DN activation and connectivity patterns^11^,^12^,^13^. These DN brain modulations typically reflect a relative *failure* to deactivate PMC during memory encoding^8^, a pattern that may explain the poor episodic memory characteristic of AD^14^.

These findings, as well as the notable topographic overlap between DN alterations and PMC Aβ deposition^15^,^16^, have lead to proposals that extended periods of high brain activity in PMC may accelerate Aβ deposition, which leads to neural dysfunction, and the onset of later life episodic memory deficits^15^,^17^. Consistent with this, functional neuroimaging (fMRI) in young APOE-ε4 carriers, who are unlikely to have Aβ deposition^18^, has revealed early alterations in PMC and medial temporal lobe regions during episodic memory^19^,^20^, and at rest^21^, relative to APOE-ε4 non-carriers.

The significance of these early-life, APOE-ε4-associated brain changes is unclear^17^; in particular, our knowledge of the contexts under which they can be elicited, and the extent to which they mirror the earliest cognitive changes in AD, is limited. Here, we provide new knowledge relevant to this question by asking whether healthy young adult APOE-ε4 carriers would show BOLD modulations in PMC for scenes, but not faces or objects, during short-term memory (*Task A*), prior to asking whether—in the PMC regions-of-interest (ROIs) identified in *Task A*—a similar BOLD activity profile across scene and non-scene conditions would be evident on a trial-unique odd-one-out perceptual discrimination paradigm (*Task B*). Previous studies have demonstrated selective deficits in early AD on analogous tasks, including poor scene odd-one-out judgements^22^, impaired same/different scene discriminations^23^, and deficient short-term memory for scene stimuli^24^. It has never been demonstrated, however, that this clinically relevant, functionally selective, neurocognitive profile can be elicited using neuroimaging methods in young adults who are at increased risk of later life AD via the presence of a major risk gene allele (APOE-ε4). Based on the published clinical studies, we predicted BOLD modulations in PMC on scene, but not face and object, conditions in both paradigms in our young APOE-ε4 carriers. This pattern would not be evident in our pair-wise matched (by age, gender, education and family history) APOE-ε4 non-carrier group.

## Results

### Behavioural Analysis

#### Task A

In *Task A*, our one-back visual working memory task, behavioural performance was calculated as hits (correct identification of a repetition) minus false alarms (FA, incorrect response to non-repeated item, see Table 1). Values were submitted to a two-way mixed model ANOVA, comprising a between-subjects factor of Group (APOE-ε4 carriers; APOE-ε4 non-carriers), and a within-subjects factor of Stimulus (scenes; faces; objects; scrambled objects). This revealed a main effect of Stimulus (*F*(3, 84) = 20.03, *p* < 0.0001, η^2^_p_ = 0.42, 95% CI [0.24, 0.53]); but no effect of Group nor a significant interaction (all *p*s > 0.14). To investigate the main effect, paired-sample t-tests (two-tailed) comparing performance between individual stimulus conditions, corrected for multiple comparisons (Bonferroni correction; α = 0.05/6 = 0.008), were undertaken. There was greater discrimination accuracy for objects and scenes relative to faces (*t*(29) = 6.59, *p* < 0.0001, and *t*(29) = 4.36, *p* < 0.0001, respectively), and scrambled objects (*t*(29) = 6.29, *p* < 0.0001, and *t*(29) = 3.94, *p* = 0.0005, respectively). Discrimination accuracy for objects and scenes (*t*(29) = 2.29, *p* = 0.03), and for faces and scrambled objects (*t*(29) = 0.88, *p* = 0.38) did not differ.

#### Task B

In *Task B*, involving the visual odd-one-out paradigm, proportion correct and mean reaction time for each condition were submitted to a two-way mixed model ANOVA including a between-subjects factor of Group (APOE-ε4 carriers; APOE-ε4 non-carriers) and a within-subjects factor of Stimulus (scenes; faces; objects; squared blocks; Table 1). The Group*Stimulus mixed model ANOVA revealed no main effects, and neither was there an interaction (all *p*s > 0.19). Reaction times were matched across groups, and again there was no interaction (all *p*s > 0.18). There was a main effect of Stimulus (*F*(3, 69) = 54.14, *p* < 0.0001, η^2^_p_ = 0.70, 95% CI [0.57, 0.77]). Paired sample t-tests (two-tailed), corrected for multiple comparisons (Bonferroni correction; α = 0.05/6 = 0.008) revealed quicker responses to squared block relative to face (*t*(24) = 7.80, *p* < 0.0001), object (*t*(24) = 10.27, *p* < 0.0001) and scene (*t*(24) = 10.28, *p* < 0.0001) trials.

### MRI Analysis

#### Task A

**Figure 1:**
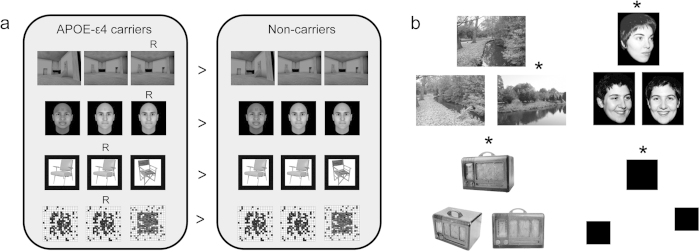
(**a**) A schematic of the one-back visual working memory task used in *Task A*. This comprised four conditions, scenes, faces, objects, and scrambled objects, and required participants to indicate when there was an immediate repeat of an item (indicated here by ‘R’). Activity for each separate stimulus condition was contrasted between APOE-ε4 carriers and APOE-ε4 non-carriers to identify regions within posteromedial cortex (PMC) sensitive to APOE-ε4 status. Scenes were created using Deus Ex (Ion Storm L.P., Austin, TX, USA, with software development package Deus Ex Software Development Kit v1112f); faces were generated with FaceGen Modeller 3.3 (Singular Inversions Inc., Toronto, ON, Canada); the objects shown in the figure are examples of the types of trials used in the task and are taken from the Hemera Photo-Objects 50,000, Volumes 1–3. (**b**) Examples of scene, face, object and squared blocks (baseline) odd-one-out trials from *Task B* (asterisks indicate the odd item). Scene photographs were taken by J.P.S.; faces are part of the Psychological Image Collection at Stirling (PICS. http://pics.stir.ac.uk/); objects are from the Hemera Photo-Objects 50,000, Volumes 1–3.

**Figure 2:**
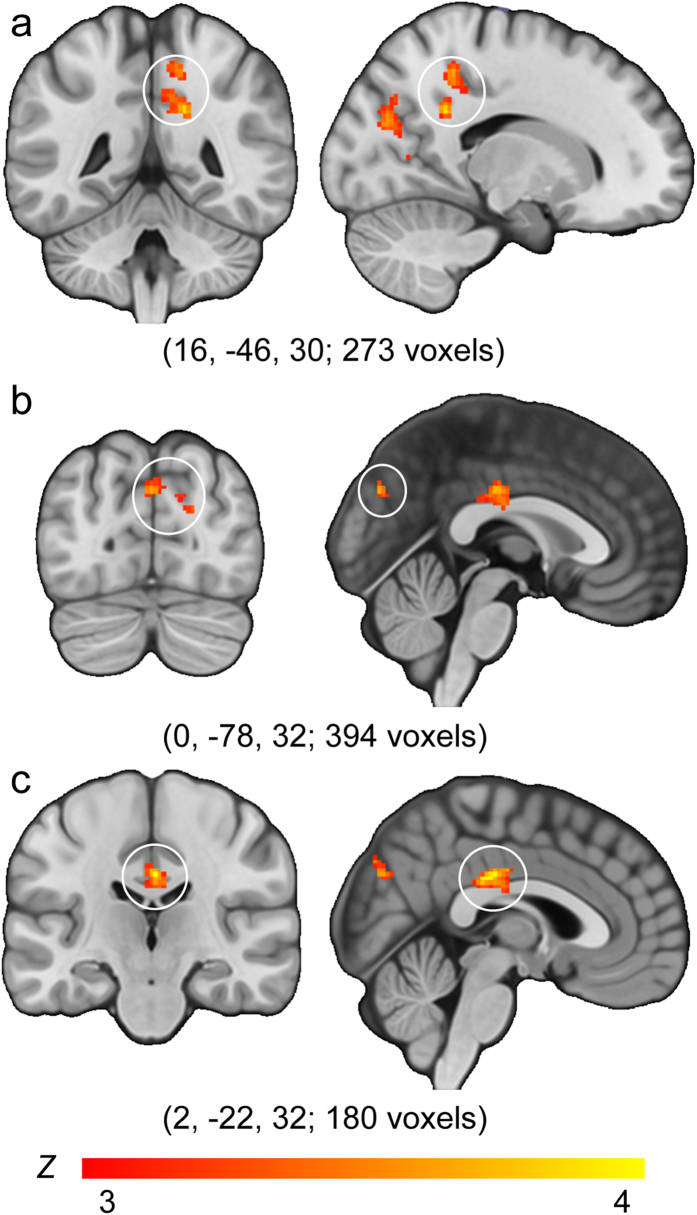
In the one-back visual working memory task (*Task A*), a selective group difference between APOE-ε4 carriers versus non-carriers in the scene condition (constrained to an *a priori* PMC ROI) was evident in (**a**) posterior cingulate cortex (PCC), (**b**) precuneus, and (**c**) cingulate. For the analyses undertaken in *Task B*, these significant clusters were binarised to generate independent regions-of-interest (ROIs). For information, in *Task B*, there were no clusters that survived the whole-brain threshold used in *Task A* (Z > 3.1, p > 0.05). All statistical maps are presented on the MNI152 template image.

In *Task A*, we compared the BOLD response elicited during the one-back visual working memory task in APOE-ε4 heterozygote carriers versus APOE-ε4 non-carriers for scenes, faces, objects and scrambled objects (Fig. 1a). Based on our *a priori* hypotheses, whole brain activation maps were intersected with a PMC ROI^25^ (see Methods). Consistent with the neurocognitive patterns reported in established AD^22^,^23^,^24^, significant group differences (APOE-ε4 carriers > non-carriers) were seen in posterior cingulate cortex (PCC; peak MNI coordinates 16, −46, 30, Z max = 3.95), precuneus (0, −78, 32, Z max = 3.66) and cingulate (2, −22, 32, Z max = 3.99) in the *scene* condition only (Fig. 2).

#### Task B

To avoid circular analysis^26^ and provide an unbiased measure of the APOE-ε4 effect size on scene processing, we used the independently defined PMC ROIs identified in *Task A* to explore the separate fMRI dataset obtained in *Task B*. In *Task B*, the same participants made odd-one-out judgements to trial-unique three-choice arrays containing scenes, faces, objects, or squared blocks (Fig. 1b). This task elicits deficits in AD patients for scene discrimination only^22^. Percentage signal change for scenes, faces and objects versus squared blocks was extracted from the three clusters identified in *Task A*.

**Figure 3:**
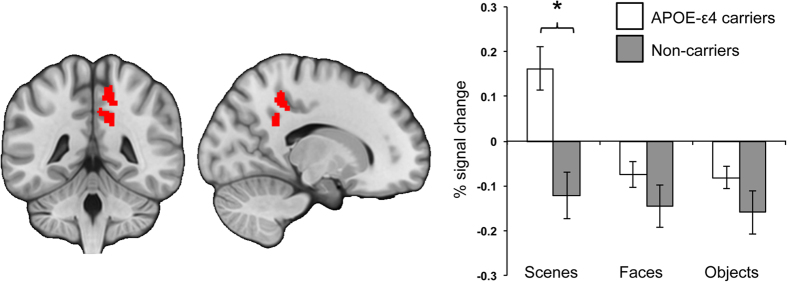
In the independently defined PCC ROI (left figure), extracting percentage signal change values for each odd-one-out condition separately compared to the squared blocks odd-one-out baseline revealed significantly greater activity (i.e., a failure to deactivate PCC) in APOE-ε4 carriers relative to APOE-ε4 non-carriers for scenes only (right figure). *p = 0.001 (Bonferroni-corrected critical 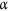 = 0.017).

In *Task B*, PCC showed a main effect of Group (*F*(1, 23) = 8.11, *p* = 0.01, η^2^_p_ = 0.26, 95% CI [0.02, 0.49]) and Stimulus (*F*(2, 46) = 15.86, *p* < 0.001, η^2^_p_ = 0.41, 95% CI [0.17, 0.55]). A significant interaction (*F*(2, 46) = 9.35, *p* < 0.001, η^2^_p_ = 0.29, 95% CI [0.07, 0.45]) confirmed statistically different activity profiles across conditions in APOE-ε4 carriers (*F*(2, 24) = 26.53, *p* < 0.001, η^2^_p_ = 0.69, 95% CI [0.41, 0.79]), but not non-carriers (*F*(2, 22) = 0.45, *p* = 0.65, η^2^_p_ = 0.04, 95% CI [0.04, 0.24]). APOE-ε4 carriers showed significantly greater activity associated with scenes relative to both faces (*t*(12) = 5.24, *p* < 0.001) and objects (*t*(12) = 5.59, *p* < 0.001); activity for faces and objects did not differ (*t*(12) = 0.34, *p* = 0.740, Fig. 3). Pair-wise comparisons confirmed greater activity (a relative lack of deactivation compared to baseline) in APOE-ε4 carriers versus non-carriers for scenes (*t*(23) = 4.01, *p* = 0.001, *d* = 1.61, 95% CI [0.70, 2.51], scaled-information B_10_ = 71.2), but not faces (*t*(23) = 1.30, *p* = 0.21, *d* = 0.52, 95% CI [−0.28, 1.32], scaled-information B_01_ = 1.3) or objects (*t*(23) = 1.47, *p* = 0.16, *d* = 0.59, 95% CI [−0.21, 1.39], scaled-information B_01_ = 1.1). The significant PCC BOLD increase in APOE-ε4 carriers for scene, but not face and object, odd-one-out discrimination is particularly striking given that behavioural performance was matched across experimental conditions and between groups (Table 1).

**Figure 4:**
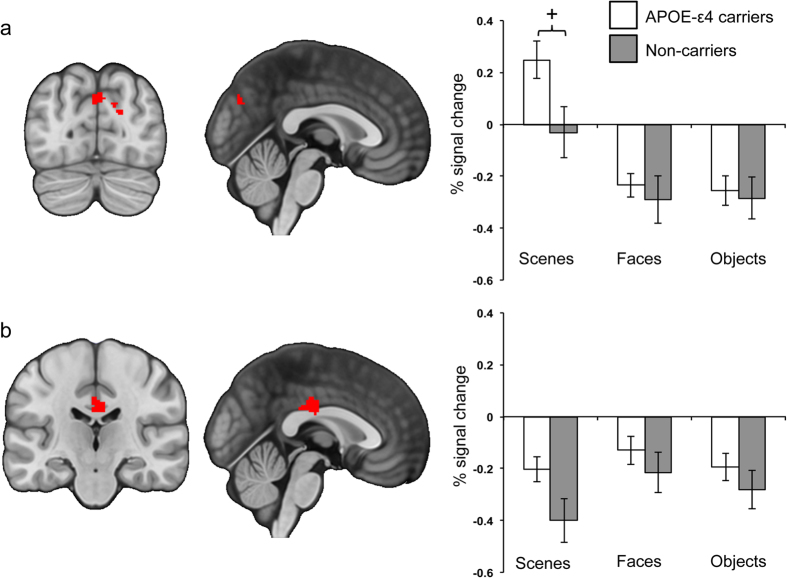
Percentage signal change values extracted from (**a**) the precuneus and (**b**) the cingulate ROIs in the scene, face and object conditions of the odd-one-out paradigm (compared with the squared blocks odd-one-out baseline). ^+^*p* = 0.03 (Bonferroni-corrected critical 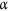 = 0.017).

In the precuneus ROI, there was a main effect of Stimulus (*F*(2, 46) = 36.36, *p* < 0.0001, η^2^_p_ = 0.61, 95% CI [0.41, 0.71]) and a Group*Stimulus interaction (*F*(2, 46) = 3.75, *p* = 0.03, η^2^_p_ = 0.14, 95% CI [0, 0.30]). Both APOE-ε4 carriers and non-carriers showed a main effect of Stimulus (*F*(2, 24) = 33.63, *p* < 0.0001, η^2^_p_ = 0.74, 95% CI [0.49, 0.82]; *F*(2,22) = 7.93, *p* = 0.003, η^2^_p_ = 0.42, 95% CI [0.08, 0.60], respectively), with greater activity associated with scenes relative to objects and faces; APOE-ε4 carriers (scenes > faces, *t*(12) = 8.28, *p* < 0.0001; scenes > objects, *t*(12) = 5.27, *p* = 0.0002; objects = faces, *t*(12) = 0.5, *p* = 0.63); APOE-ε4 non-carriers (scenes > faces, *t*(11) = 3.55, *p* = 0.005; scenes > objects, *t*(11) = 2.95, *p* = 0.01; faces = objects, *t*(11) = 0.09, *p* = 0.93). Similar to the pattern of data in PCC, the interaction in precuneus stemmed from between-group differences in scene-related activity. Although APOE-ε4 carriers showed greater activity associated with scenes relative to non-carriers, unlike PCC, this difference did not survive Bonferroni correction (*t*(23) = 2.33, *p* = 0.03, *d* = 0.93, 95% CI [0.11, 1.76], scaled-information B_10_ = 3.2); activity for faces (*t*(23) = 0.56, *p* = 0.58, *d* = 0.22, 95% CI [−0.56, 1.01], scaled-information B_01_ = 2.3) and objects (*t*(23) = 0.28, *p* = 0.78, *d* = 0.02, 95% CI [−0.76, 0.81], scaled-information B_01_ = 2.6) did not differ between groups (Fig. 4a). In the cingulate, the only significant effect was of Stimulus (*F*(2, 46) = 5.73, *p* = 0.006, η^2^_p_ = 0.20, 95% CI [0.02, 0.37]; all other *p*s > 0.15), which reflected greater activity associated with faces relative to scenes (*t*(24) = 3.87, *p* = 0.001). There was no statistical difference between the percentage signal change associated with objects and faces (*t*(24) = 1.60, *p* = 0.122), or objects and scenes (*t*(24) = 1.49, *p* = 0.15, see Fig. 4b). Note, these findings remained when the male participants were removed from analyses.

## Discussion

In *Task A*, in which participants pressed a button when they noticed the immediate repeat of a scene, object, face or scrambled object, we found a significant difference between our APOE-ε4 carrier and non-carrier groups in PMC regions (i.e., PCC, precuneus and cingulate) in the *scene* condition only. *Task B*, where participants made odd-one-out decisions to trial-unique triads of scenes, objects, faces and squared blocks, allowed further investigation of this group difference, critically in an independent cognitive paradigm stressing complex perceptual discrimination, and sensitive to scene discrimination deficits in clinical AD^22^. Consistent with the behavioural finding of impaired scene, but not face, odd-one-out judgements in AD, PCC activity in young adult APOE-ε4 carriers, but not non-carriers, was significantly altered on this perceptual task for scenes, relative to faces and objects. Two aspects of this latter finding are novel. First, that PMC BOLD changes in young APOE-ε4 carriers, or more explicitly a relative failure to deactivate PCC compared to non-carriers, can be elicited during perceptual discrimination, and second, that these changes are particularly evident when individuals are asked to differentiate between complex scenes, relative to difficulty-matched faces and objects.

Our unique findings, in predominantly female college-aged individuals, strengthen accounts in which AD risk is best understood by considering a life-course approach^17^, specifically, where an inability to modulate posterior DN activity—present over many years—may eventually precipitate AD pathology and cognitive decline^15^. This view predicts that topographically, and functionally-specific, brain alterations should be evident in at-risk individuals in advance of Aβ deposition, a hypothesis confirmed here where our young adult APOE-ε4 participants—highly unlikely to be Aβ-positive^18^—showed a failure to deactivate PCC, but only when they were performing scene perception and scene working memory tasks. In fact, during scene odd-one-out, the APOE-ε4 carriers actually showed a positive MRI signal response in PCC. This pattern mirrors results in older cognitively healthy APOE-ε4 individuals who do not yet show significant Aβ deposition^27^, as well as findings in pre-symptomatic older individuals with a positive Aβ status^8^,^27^,^28^ or poor episodic memory^29^. As it has also been demonstrated that Aβ levels in older individuals are positively associated with their APOE-ε4 status^30^, it seems plausible to assume that there may be a critical association between APOE-ε4 and early life posterior DN changes, which in turn could influence the spatial distribution of Aβ deposition in key brain regions implicated in preclinical AD^15^,^16^. Consistent with this, recent studies in autosomal dominant AD have also identified striking DN-related posteromedial changes prior to Aβ deposition and the onset of cognitive decline^31^.

Further support for this account comes from transgenic mice studies^32^,^33^. For example, in APP transgenic mice expressing a mutated form of Aβ precursor protein, lactate (as a measure of neuronal activity) was closely associated with interstitial fluid (ISF) Aβ levels, and in turn, ISF Aβ predicted region-specific Aβ deposition, particularly in DN brain areas^32^. In a further transgenic mouse study, a striking link between reduced bilateral functional connectivity in PMC and Aβ deposition was demonstrated^33^, a pattern that has also been reported in cognitively normal older participants^9^,^34^ and in preclinical AD^35^. It has been proposed that the APOE-ε4 genotype contributes to the hypothesised activity-dependent process promoting later life Aβ deposition by reducing neural efficiency^17^. PMC may be particularly vulnerable to the effects of reduced neural efficiency, due to its continual ‘toggling’ between rest-induced high activity and task-related deactivation^8^,^15^,^36^, as well as its role as a large-scale connectivity hub^3^. The mechanism by which APOE alters neural efficiency, however, is not yet clear; an answer to this question may come via study of APOE-ε4’s influence on synaptic plasticity/repair, neurovasculature, lipid metabolism, and/or neurodevelopment (see ref. 37, for review).

Here, with the aim of generating new knowledge relevant to understanding the specificity of early-life DN brain alterations, we tested the hypothesis that scene processing may be a particularly sensitive AD-relevant endophenotype. This prediction stems from an emerging focus within the memory field on accounts that blur the neural distinction between perception and memory, instead highlighting the importance of domain-specific representational information able to be utilised, in a goal-directed manner, across different cognitive domains (e.g., perception, short-term memory and episodic memory)^38^. In such views, complex spatial representations function as a critical scaffold for successful episodic memory^38^, a conclusion supported by evidence that the functional brain networks involved in episodic memory and spatial navigation show significant topographical correspondence^3^,^39^. These brain networks also partially overlap with PMC DN activations seen during rest and minimally demanding tasks^3^. With regard to episodic memory in particular, imaging studies typically show PMC deactivations during memory encoding that are associated with PMC activations seen at retrieval^4^. The reliable group difference seen between APOE-ε4 carriers and non-carriers, localised to PCC and selective to scene processing, across two different cognitive paradigms (one-back working memory and perceptual discrimination), is consistent with this literature, and with the representational accounts of memory outlined above.

Our findings are particularly valuable when considered alongside data that AD patients show selective deficits on a scene, but not face, odd-one-out task similar to that used here^22^; notably, these impairments extend to same/different scene discriminations^23^ and short-term memory for scene stimuli^24^. The striking congruency between these *later-life* cognitive deficits, and our *early-life* APOE-ε4-influenced PCC modulations, supports the idea that poorer cognitive health in older age may reflect a—potentially predictable—sequence of functionally selective neural changes experienced across the lifespan; individual susceptibility to these brain changes is most likely influenced by key lifestyle and genetic factors, including APOE-ε4 status^17^.

Although our APOE-ε4 carrier and non-carrier groups were pair-wise matched for gender (amongst other key factors), the data were obtained in a predominantly female sample. Notably, prior research has determined that the detrimental impact of the APOE-ε4 allele on AD risk^40^, Aβ pathology^41^ and PMC resting connectivity^42^ is most pronounced in women. It is possible, therefore, that the sizeable DN modulations evident in our sample on scene tasks may be attenuated in male carriers of the APOE-ε4 allele. Testing for potential moderators of APOE-ε4’s influence on brain activity (e.g., gender) will require a move towards larger, population representative, samples, going beyond smaller-scale studies^20^,^43^, including ours, where power to detect such interactions is limited (see ref. 44).

In conclusion, our study reveals that detailed assessment of complex scene processing and mnemonic ability in young individuals at increased risk of AD may provide a unique window into the genesis of poorer later-life cognitive health. Our odd-one-out task, for example, is an easily administered paradigm able to measure scene discrimination ability across the life-span, as well as facilitate cross-species translational approaches to cognitive aging^45^. Of course, we do not yet know whether our early-life functional DN modulations are definitively predictive of poorer later-life cognitive health, and nor do we know when, and how, APOE-ε4 may contribute to pathological versus non-pathological cognitive aging^46^,^47^. Answering these questions is the critical next step in testing hypotheses about the time course of brain changes that may underpin increased risk, and early onset, of AD.

## Materials and Methods

### Participants

College-age Psychology students (n = 125) provided a saliva sample for DNA extraction and APOE genotype status. APOE genotyping was carried out using Applied Biosystems, Assay-on-demand TaqMan^®^ SNP Genotyping Assays, C_3084793_20 and C_904973_10 corresponding to APOE single nucleotide polymorphisms (SNPs) rs429358 and rs7412, respectively, and run on a LJL Biosystems Analyst HTS Assay Detection Platform. Haplotypes corresponding to APOE-ε2, -ε3 and -ε4 were then deduced. Genotyping was successful in 100 participants. APOE-ε4 is a strong genetic risk factor for sporadic AD, with dependent lifetime risks at 85 years estimated as 51–60% (APOE-ε4ε4) and 23-30% (APOE-ε3ε4), relative to 7–10% for APOE-ε3ε3 genotype^10^. The genotypic distribution of those successfully genotyped was ε2ε2 (1/100, 1%), ε2ε3 (10/100, 10%), ε2ε4 (1/100, 1%), ε3ε3 (69/100, 69%), ε3ε4 (19/100, 19%), and ε4ε4 (0/94, 0%). The observed APOE genotypic distribution in our population closely matches the expected frequencies in the normal population (χ^2^ = 4.48, *df* = 5, *p* = 0.48)^48^. Based on the presence/absence of an APOE-ε4 allele, two groups (20 participants in each) were created. Individuals in these groups were *pair-wise* matched for gender, educational level and age.

Due to attrition resulting from scanning non-attendance, MRI contraindications and withdrawal during testing, the available sample for analysis in *Task A* was 15 participants in each group (a sample size similar to other studies in the literature, e.g., see refs 20,43). The non-carrier APOE allele distribution was 10 APOE-ε3ε3 and 5 APOE-ε2ε3 individuals. The carrier APOE allele distribution was 14 APOE-ε3ε4 and 1 APOE-ε2ε4. Given our pair-wise matching approach, and the homogeneous nature of the population from which we selected (first year undergraduate students), the mean age for the two groups reported in *Task A* was identical (APOE-ε4 carriers: 19.7 years; S.D. = 0.84, APOE-ε4 non-carriers: 19.7 years; S.D. 0.89). Similarly, all participants were at the same higher educational level. The 30 participants comprised 28 females (14 in each group), and 2 males (1 in each group), a distribution reflecting the use of a predominantly female Psychology student cohort.

Family history was also matched across the two groups, with two reports of a positive family history in both (e.g., 13% of each group). In the APOE-ε4 non-carrier group, there was one positive report of Parkinson’s disease (in a great uncle aged approximately 75 years) and a report of dementia (variety not known) in a grandmother (in their 60s). In the APOE-ε4 carrier group, there was also a report of Parkinson’s disease in a grandmother (aged in their late 50s), and one report of dementia (variety not known) in a great grandmother (aged 90 years).

All participants were right-handed, native-English speakers with normal or corrected-to-normal vision, and no self-reported history of neurological or psychiatric disorders (confirmed using the MINI)^49^. This research was conducted in a double-blind manner whereby participants, and researchers collecting and analysing data, were blind to APOE status. All experimental procedures were conducted in accordance with, and were approved by, the Cardiff University School of Psychology Research Ethics Committee. Informed consent was obtained from all participants.

### Standard Neuropsychological Assessment

Standard screening questionnaires, episodic memory and executive function tests were given to participants prior to imaging; as shown in Table 2, the groups were well matched (all *t*s < 1.90, *p*s > 0.05; two-tailed), with a small advantage (between 1–2 points) in the APOE-ε4 carriers in the semantic association Camel and Cactus Test (*t*(28) = 3.21, *p* = 0.003; two-tailed).

### Experimental  Tasks

Two experimental tasks were used in the study. All participants completed *Task A* before *Task B*.

#### Task A (One-back visual working memory task)

Participants viewed sequentially presented computer generated scenes (created using Deus Ex, Ion Storm L.P., Austin, TX, USA, with software development package Deus Ex Software Development Kit v1112f), faces (generated with FaceGen Modeller 3.3, Singular Inversions Inc., Toronto, ON, Canada), objects (chairs from Hemera object database Vol. 1–3) and scrambled objects, and responded with a button press when they saw an immediate item repeat (Fig. 1a). There were 32 exemplars in the scene, face and object conditions; 16 for scrambled objects. All stimuli were different to those used in *Task B*. During the task 192 items per class were displayed, drawn randomly from their respective sets. Items were presented in a blocked design, with 16 items per class presented per block. One-back targets (i.e., immediate repetitions) occurred randomly across the experimental task (so the frequency of targets was not predictable within blocks), and could vary across participants. The number of repeats was matched across APOE groups and stimulus categories (ps > 0.2). Images were shown for 200 ms, with a 800 ms inter-stimulus interval (ISI). There were 52 blocks in total. Blocks 1, 18, 35, and 52 comprised baseline fixation crosshairs to allow the participant to prepare for experimental blocks. Blocks 2—17 followed the sequence objects/faces/scenes/scrambled objects (with this sequence repeated four times); blocks 19–34 the sequence, scrambled objects/scenes/faces/objects (again repeated four times); and blocks 36–51, scrambled objects/faces/scenes/objects (repeated four times). Images were shown using Presentation software (Neurobehavioral Systems, Albany, California, USA) software at a size of 400 by 400 pixels.

#### Task B (Odd-one-out task)

Three images from the same category were presented concurrently, positioned top middle, bottom left and bottom right (Fig. 1b). There were four classes of stimuli: scenes (real world vistas photographed by the experimenters), novel faces^50^, objects (acquired from Hemera object database, Vol. 1–3), and squared blocks. For the scenes, faces and objects, two of the images comprised the same item presented from different viewpoints, whilst the third was a perceptually similar, but different, item. In the squared block condition (baseline), two of the squared blocks were the same size whilst the third differed in size by 9–15 pixels^50^. Participants identified the odd-one-out using an MR compatible button-box. The task was administered in the scanner over three functional imaging runs. There were six mini-blocks per run, each of which comprised three trials of each stimulus class (scenes, faces, objects, squared blocks) presented sequentially. Participants were randomly assigned to one of four different task versions (which counterbalanced the order of stimulus class mini-blocks). Each trial was presented for 6 seconds with a jittered inter-trial interval of 500–4000 ms. Overall, 18 trials were presented per category per run resulting in 54 trials per condition overall. An equal number of targets appeared at each screen position (i.e., top centre; bottom left; bottom right) for each stimulus class. Stimuli were presented in the scanner using ePrime (Psychology Software Tools, Inc., Sharpsburg, PA) and projected onto the screen behind the participant using a Canon SX60 LCOS projector system combined with a Navitar SST300 zoom converter lens.

### MRI Scanning Parameters

Data were collected at the Cardiff University Brain Research Imaging Centre (CUBRIC) using a GE 3-T HDx MRI system with an 8-channel receive-only head coil. An EPI pulse sequence was used to acquire T2*-weighted image volumes with BOLD contrast (TR/TE = 3000/35 ms, FOV = 240 mm, 64*64 data matrix, and ASSET (acceleration factor), 90° flip angle). Forty-two slices were collected in an interleaved fashion per image volume for whole brain coverage. Each slice was 2.4 mm thick with a 1mm inter-slice gap (3.4*3.4*2.4 mm voxels). Slices were acquired with a 30° axial-to-coronal tilt relative to the AC-PC line (anterior upwards). The first four volumes of each scanning run were discarded to allow for signal equilibrium. Two 3D SPGR images were acquired at the beginning of the first scanning session to improve registration and reduce image distortion as a result of magnetic-field inhomogeneity (TE = 7 ms and 9 ms, TR = 20 ms, FOV = 384 * 192 * 210 mm, 128 * 64 * 70 data matrix, 10° flip angle). The SPGR used the same slice orientation as the EPI data. High-resolution anatomical images were acquired using a standard T1-weighted 3D FSPGR sequence comprising 178 axial slices (TR/TE = 7.8/3.0 s, FOV = 256 * 256 * 176 mm, 256 * 256 * 176 data matrix, 20° flip angle, and 1mm isotropic resolution).

### MRI Pre-processing

Imaging pre-processing was carried out using standard procedures in FEAT (FSL, www.fmrib.ox.ac.uk/fsl)^51^, and comprised motion correction, brain extraction using BET, spatial smoothing with a Gaussian kernel of FWHM 5 mm, mean-based intensity normalisation, and high-pass temporal filtering (Gaussian-weighted least-squares straight line fitting, with sigma = 100 s). Phase information from the two SPGR images was unwrapped using PRELUDE. The unwrapped phase images were then subtracted and the resulting fieldmap used to unwarp the EPI data using FUGUE. Time-series statistical analysis was carried out using FMRIB’s Improved Linear Model (FILM) with local autocorrelation correction. Registration to high resolution 3D anatomical T1 scans (per participant) and to a standard Montreal Neurological Institute (MNI152) template image (for group average) was carried out using FLIRT. Following pre-processing, analyses were first conducted at the single-subject level using FILM. The BOLD signal was modelled using a standard hemodynamic response function (HRF). Stereotactic co-ordinates of significant effects are reported in MNI space.

### Participant Exclusion Criteria

Participant data were excluded from an analysis if head movement exceeded 3mm (one voxel) during any scanning run. This affected *Task B* only, where data from four participants were removed (two from each group), as well as a further participant due to scanner error (n = 13, APOE-ε4 carriers and n = 12, APOE-ε4 non-carriers). In this group, the carrier APOE allele distribution was 12 APOE-ε3ε4 and 1 APOE-ε2ε4; the non-carrier APOE allele distribution was 8 APOE-ε3ε3 and 3 APOE-ε2ε3 individuals.

### MRI Analysis

In *Task A*, four explanatory variables (EVs), comprising scene, face, object, and scrambled object blocks, were used to model the time course data. A General Linear Model was implemented to examine the BOLD response associated with each separate stimulus class compared to rest. Anatomical masks of PMC^25^ were used to define regions-of-interest (ROIs) by calculating group averages (for APOE-ε4 carriers and non-carriers) for each stimulus class, and then undertaking contrasts between groups. Group analyses were carried out using the FMRIB Local Analysis of Mixed Effects tool (FLAME). To account for possible between-group differences in brain structure, individual grey matter (GM) images were entered into the group-level model as a voxel-wise covariate using FMRIB’s Automated Segmentation Tool (FAST)^52^. In FEAT, a general linear model was used to create two groups on the basis of APOE status. The activity associated with each stimulus class separately was then compared between the two participant groups. The resulting group-difference Z-statistic images were thresholded whole brain for clusters surviving Z > 3.1; a family-wise error (FWE) corrected cluster extent of *p* < 0.05 was applied, based on the theory of Gaussian Random Fields. Finally, whole brain Z-statistic images were intersected with an anatomical mask of the PMC, as defined by previous studies (e.g., see ref. 25). This anatomical PMC mask was created by combining probabilistic masks (thresholded at 50%) of the PCC and precuneus cortex from the Harvard-Oxford cortical structural atlas.

In *Task B,* the three experimental runs were modelled separately, with five EVs comprising correct responses to scene, face, object and squared block trials, and one regressor comprising all incorrect responses. The duration of the regressors corresponded to the oddity trial duration (i.e., 6 seconds). Scenes, faces and objects were all contrasted with squared blocks (baseline). The three individual runs for each participant were then combined using a fixed effects model. Percentage signal change values for the three contrasts (scenes compared to squared blocks; faces compared to squared blocks; objects contrasted with squared blocks) were extracted from cluster-based ROIs (see Fig. 2) derived from *Task A* and entered into separate mixed model ANOVAs comprising a between-subjects factor of Group (APOE-ε4 carriers; APOE-ε4 non-carriers), and a within-subjects factor of Stimulus (scenes; faces; objects). A significant interaction between these factors was investigated with separate one-way, repeated-measures ANOVAs examining the profile of BOLD activity for individual stimulus classes within each APOE group. Significant main effects were investigated using paired-sample t-tests (two-tailed) comparing the percentage signal change associated with the three stimulus classes (Bonferroni correction; α = 0.05/3 = 0.017). To examine whether the interaction stemmed from BOLD activity differences across the two participant groups, independent sample t-tests (two-tailed) were used to compare percentage signal change values for each stimulus class across the two groups (Bonferroni correction; α = 0.05/3 = 0.017).

For key contrasts of interest, we also performed complementary Bayesian analyses. Bayes factors indicate the relative strength of evidence for two theories^53^. The Bayes factor (B) comparing the alternative hypothesis to the null hypothesis (B_10_) means that the data are B times more likely under the alternative than the null, and *vice versa* (B_01_). B_10_ values much greater than 1 allow us to draw the conclusion that there is strong evidence for the alternative over the null. Similarly, B_01_ values much greater than 1 support the conclusion that there is strong evidence in favour of the null^53^. Unit-information Bayes factors were calculated using an online Bayes factor calculator^53^ (http://www.pcl.missouri.edu/bayesfactor).

## Additional Information

**How to cite this article**: Shine, J. P. *et al.* APOE-ε4 selectively modulates posteromedial cortex activity during scene perception and short-term memory in young healthy adults. *Sci. Rep.* **5**, 16322; doi: 10.1038/srep16322 (2015).

## Figures and Tables

**Table 1:** Behavioural performance (means and standard deviations) in the one-back visual working memory and odd-one-out tasks: data are split by condition and by APOE group.

	APOE-ε4 carriers	Non-carriers
***Task A*****: Visual working memory task**	*n* = *15*	*n* = *15*
Mean hit minus FA rate (S.D.)		
Scenes	0.75 (0.12)	0.61 (0.21)
Faces	0.55 (0.19)	0.52 (0.22)
Objects	0.78 (0.12)	0.70 (0.23)
Scrambled objects	0.57 (0.23)	0.56 (0.19)
***Task B*****: Odd-one-out task**	*n* = 13	*n* = 12
Mean proportion correct (S.D.)		
Scenes	0.85 (0.06)	0.86 (0.07)
Faces	0.89 (0.08)	0.88 (0.07)
Objects	0.87 (0.05)	0.83 (0.08)
Squared blocks	0.82 (0.12)	0.86 (0.13)
Mean reaction time (S.D., ms)		
Scenes	2629 (586)	2809 (321)
Faces	2505 (424)	2582 (249)
Objects	2648 (447)	2635 (275)
Squared blocks	1944 (407)	2163 (312)

There were no significant differences, on any measure, between the APOE-ε4 carrier and APOE-ε4 non-carrier groups.

**Table 2:** Mean scores and standard deviations (in parentheses) for each of the background neuropsychology tasks are shown, split by APOE group.

	APOE-ε4 carriers	Non-carriers
Memory Recall^54^,^55^		
WMS III immediate story recall (/75)	40.27 (9.04)	41.53 (6.80)
WMS III delayed story recall (/50)	27.73 (7.37)	26.93 (5.36)
RCF—delayed recall (/36)*	26.47 (5.46)	24.81 (6.22)
Recognition Memory		
WMS III delayed story recognition (/30)	26.2 (2.78)	26.67 (1.76)
Visuospatial Processing		
RCF—copy (/36)*	35.40 (0.91)	35.23 (1.01)
Semantic Memory^56^		
CCT (/64)^∆^	58.53 (2.59)	56.64 (1.74)
Executive Function^57^,^58^		
RPCM (/36)	34.33 (1.11)	32.67 (1.67)
D-KEFS—Visual scanning (secs)*	10.80 (1.47)	10.53 (1.27)
D-KEFS—Number sequencing (secs)*	11.00 (1.13)	9.92 (1.85)
D-KEFS—Letter sequencing (secs)*	11.33 (1.50)	10.54 (3.07)
D-KEFS—Number-letter switching (secs)*	11.20 (0.86)	10.54 (1.85)
D-KEFS—Motor speed (secs)*	11.20 (1.21)	10.92 (1.44)

Table abbreviations refer to the Wechsler Memory Scale (WMS), Rey Complex Figure (RCF), Camel and Cactus Test (CCT), Raven’s Colored Progressive Matrices (RCPM) and the Delis–Kaplan Executive Function System (D-KEFS). The only significant difference between the APOE-ε4 carrier and APOE-ε4 non-carrier groups was on the Camel and Cactus Test; this was a small (1–2 point) advantage in the APOE-ε4 carrier group.

^*^scores based on 15 APOE-ε4 carriers and 13 non-carriers; ∆ scores based on 15 APOE-ε4 carriers and 14 non-carriers. The lack of data from 1 or 2 individuals in the APOE-ε4 non-carrier groups reflected attrition in testing compliance near the end of the multiple testing sessions undertaken in this study.
